# A Reduced Order Model for Sea Water Intrusion Simulation Using Proper Orthogonal Decomposition

**DOI:** 10.1111/gwat.13462

**Published:** 2024-12-27

**Authors:** Mohammadali Geranmehr, Domenico Bau, Alex S. Mayer, Weijiang Yu

**Affiliations:** ^1^ Department of Civil and Structural Engineering The University of Sheffield Sheffield UK; ^2^ University of Texas at El Paso El Paso Texas

## Abstract

Sea water intrusion (SWI) simulators are essential tools to assist the sustainable management of coastal aquifers. These simulators require the solution of coupled variable‐density partial differential equations (PDEs), which reproduce the processes of groundwater flow and dissolved salt transport. The solution of these PDEs is typically addressed numerically with the use of density‐dependent flow simulators, which are computationally intensive in most practical applications. To this end, model surrogates are generally developed as substitutes for full‐scale aquifer models to trade off accuracy in exchange for computational efficiency. Surrogates represent an attractive option to support groundwater management situations in which fast simulators are required to evaluate large sets of alternative pumping strategies. Reduced‐order models, a sub‐category of surrogate models, are based on the original model equations and may provide quite accurate results at a small fraction of computational cost. In this study, a variable‐density flow reduced‐order model based on proper orthogonal decomposition (POD) and utilizing a fully coupled flow and solute‐transport model is implemented with a finite‐difference (FD) approach for simulating SWI in coastal aquifers. The accuracy and computational efficiency of the FD‐POD approach for both homogeneous and—more realistic—heterogeneous systems are investigated using test cases based on the classic Henry's problem (Henry 1964). The findings demonstrate that the combined FD‐POD approach is effective in terms of both accuracy and computational gain and can accommodate the output of the most popular variable‐density flow models, such as those from USGS's MODFLOW family.

## Graphical abstract

In this study, a variable‐density flow reduced‐order model based on proper orthogonal decomposition (POD) and utilizing a fully coupled flow and solute‐transport model is implemented with a finite‐difference (FD) approach for simulating sea water intrusion (SWI) in coastal aquifers.

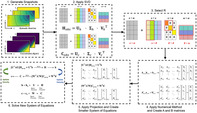

## Introduction

Coastal regions globally face mounting challenges in managing groundwater resources. These aquifers are increasingly stressed due to escalating demand and the compounding effects of climate change, sea‐level rise, and variations in groundwater recharge patterns (Ketabchi et al. 2016; Alfarrah and Walraevens 2018; Befus et al. 2020; Panthi et al. 2022). Among the most pressing issues confronting coastal aquifers is SWI, a phenomenon where saline water infiltrates freshwater aquifers driven by density variations. This intrusion not only jeopardizes water quality but also escalates management costs and threatens the sustainability of groundwater resources.

To address the complexities of coastal groundwater systems, groundwater models have emerged as indispensable tools for water administrators and stakeholders. Models such as SUTRA (Voss 1984), MODFLOW 6 (MF6) (Langevin et al. 2017), and SEAWAT (Guo and Langevin 2002; Langevin et al. 2008), which is based on MODFLOW‐2005 (Harbaugh 2005) and MT3DMS (Zheng and Wang 1999), play a pivotal role by simulating groundwater flow and solute transport. Notably, these models are considered “coupled” variable‐density models, as the migration of salt affects density, consequently impacting flow dynamics, which in turn affects salt transport. This intricate relationship underpins the coupling between flow and transport processes in coastal aquifers.

Achieving solutions to the coupled PDEs underlying groundwater flow and solute transport relies on sophisticated numerical techniques. Discretization methods, including FD, used in SEAWAT, finite elements (FE), used in SUTRA, and finite volumes (FV), used in MF6, are employed to transform PDEs into systems of ordinary differential equations (ODEs). However, the computational demands of these models, necessitating fine grid resolution and iterative coupling, present significant challenges. For example, the computational intensity and data requirements of groundwater models often hinder their application in practical management applications. Ideally, models should balance computational efficiency with accuracy to enable repeated simulations for optimization, risk analysis, and decision support. To address these challenges, surrogate models have emerged as viable alternatives, trading off accuracy for computational speed. These models can be grouped into three main categories: data‐driven surrogates, multi‐fidelity surrogates, and model‐driven surrogates. A comprehensive review of surrogates may be found in Asher et al. (2015).

Data‐driven surrogates rely on “observations” to generate predictions based on statistical or machine‐learning techniques to learn patterns directly from the data. The definition of observations is somewhat loose in that these can be compiled from actual field data or generated from full‐scale model training runs. For example, in the case of field observations, machine learning techniques have been widely applied to water level modeling in large‐scale aquifers. These techniques include, among others, artificial neural networks (ANNs) (Coulibaly et al. 2001; Krishna et al. 2008; Mohanty et al. 2010), adaptive neuro‐fuzzy inference systems (ANFIS) (Jalalkamali et al. 2010; Emamgholizadeh et al. 2014), genetic programming (Fallah‐Mehdipour et al. 2013), and support vector machines (SVMs) (Rajaee et al. 2019). In the construction of surrogates for full‐scale models, data‐driven approaches have been widely adopted for applications in contaminant transport simulation (Shekofteh et al. 2012; Arabgol et al. 2016) and water quality simulation (Haggerty et al. 2023). These techniques have also been widely adopted for variable‐density flow models, most typically in simulation‐optimization approaches to assist coastal groundwater management (Kourakos and Mantoglou 2009; Sreekanth and Datta 2011; Ataie‐Ashtiani et al. 2014; Roy and Datta 2017; Christelis et al. 2018; Lal and Datta 2018; Ranjbar and Mahjouri 2018; Song et al. 2018; Kopsiaftis et al. 2019; Yang et al. 2021) and also to conduct sensitivity analyses for uncertainty quantification (Rajabi et al. 2015; Koohbor et al. 2019; Rajabi 2019).

Multi‐fidelity surrogate models (Asher et al. 2015; Zhou et al. 2016) are a particular category of physically based models that combine information from computationally efficient low‐fidelity numerical models (i.e., with reduced numerical resolution, larger tolerance, and/or simplified physics), with “data” from higher‐fidelity models to construct a surrogate that integrates both types of data. Multi‐fidelity surrogates have also been applied to SWI simulation (Kerrou and Renard 2010; Christelis and Mantoglou 2016; Christelis and Mantoglou 2019; Dey and Prakash 2020; Christelis 2021; Christelis and Hughes 2022).

Reduced‐order models (ROMs) constitute a third important category of physically based surrogates, which most often rely on projection methods (Asher et al. 2015) to capture the essential modes of the system dynamics to mitigate computational complexity. One of the earlier applications of projection techniques to groundwater flow models was reported by Vermeulen et al. (2004), who used State‐Space Projection and Galerkin Projection techniques based on empirical orthogonal functions (EOFs) and proper orthogonal decomposition (POD) to extract dominant modes of variability from high‐dimensional data using the Karhunen–Loève expansion (Newman 1996). Numerous studies have since highlighted the versatility of POD in engineering applications (Lu et al. 2019), and particularly for subsurface flow simulation, which makes it a powerful tool for groundwater management (Mcphee and Yeh 2008). Approaches for optimizing the selection of dominant modes (Siade et al. 2010; Di et al. 2012), implementing ROMs for Monte Carlo simulation (Pasetto et al. 2011), and estimating hydraulic conductivity spatial distributions in inverse problems (Winton et al. 2011) are among the earlier reported applications of POD for developing surrogates of groundwater flow simulators.

More recently, Boyce and Yeh (2014) applied POD for parameter‐independent model reduction in transient groundwater flow in confined aquifers. Boyce et al. (2015) extended this method to unconfined groundwater flow based on Galerkin projection and the Newton formulation of MODFLOW (Niswonger et al. 2011). Of related interest are the works of Stanko et al. (2016), who combined POD and discrete empirical interpolation methods to create a reduced model implemented within the MODFLOW framework, Ushijima and Yeh (2017), who developed system matrices in a parameterized approach for creating a reduced‐order groundwater model, and Gosses et al. (2018), who developed POD based reduced‐order models that can handle complex Dirichlet, Neumann, and Cauchy boundaries conditions. More recently, Dey and Dhar (2020) applied POD to simulate flow in randomly heterogeneous confined aquifers, Xia et al. (2020) combined POD with moment equations for Monte Carlo flow simulation, Ushijima et al. (2021) coupled POD with metaheuristic algorithms to optimize design parameters in aquifers, and Dey and Dhar (2023) integrated POD within the OpenFOAM framework (Jasak et al. 2007) for groundwater flow applications.

POD‐based model reduction techniques have been applied for subsurface solute‐transport modeling as well. For example, Li et al. (2013) used POD to construct ROMs of transient mass transport in heterogeneous media, Dehghan and Abbaszadeh (2018) used POD with local radial basis functions and differential quadrature, Rizzo et al. (2018) applied POD to model solute transport in heterogeneous porous media, and Stanko and Yeh (2019) used POD to model solute transport in the presence of nonlinear sorption. POD was used for nitrate transport simulation by Noori et al. (2020), who developed PODMT3DMS, a modified version of the popular transport model MT3DMS (Zheng and Wang 1999), and by Dehghan et al. (2022), who combined POD with a finite‐element (FE) reactive transport model under advection‐dominated flow conditions.

In the simulation of variable‐density flow in coastal aquifers, POD approaches to model reduction have received limited attention, likely due to the computational challenges posed by the coupling between the flow and the transport processes. Li and Hu (2013) applied POD to a Galerkin FE model, to simulate SWI in coastal aquifers. They used singular value decomposition (SVD) to create basis functions for reducing the model size and demonstrated that POD is a promising method for developing surrogate variable‐density models.

This study aims to further investigate to application of POD‐based projection methods for developing ROMs that can support SWI management. The coupling of POD with FD solution of the coupled flow and transport equation is proposed to address the computational challenges posed by the complex dynamics of variable‐density flow observed in coastal aquifers. The model framework is tested by focusing on the classic Henry's problem (Henry 1964), under the hypothesis of advection–diffusion salt transport in heterogeneous systems. This structured approach aims to enhance the computational efficiency of the SWI simulation while minimizing the accuracy loss with respect to the full‐scale model, thereby providing an ideal tool to assist the sustainable management of coastal groundwater resources.

This article is structured as follows: Section “Methodology” outlines the methodology devised to construct, the FD‐based variable‐density flow model, which is then reduced by means of the POD scheme. In section “Numerical Experiments,” Henry's problem is defined and solved using the FD‐POD approach, in the case of both homogeneous and heterogeneous porous media. Results of these investigations are then reported and discussed comprehensively. Finally, section “Conclusions” offers a conclusion summarizing the key findings of the study.

## Methodology

### Variable Density Flow Model

Variable‐density groundwater flow models rely on solving two coupled PDEs. The first PDE governs density‐dependent flow in saturated porous media and is written as (Langevin et al. 2008):

(1) $$\nabla \left[\rho K\left(\nabla h+\frac{\rho -\rho_{f}}{\rho_{f}}\nabla z\right)\right]=\rho S_{S}\frac{\partial h}{\partial t}+\theta \frac{\partial \rho }{\partial c}\frac{\partial c}{\partial t}-\rho_{s}q_{s}$$

where ∇ is the gradient operator (1/m), *h* is the equivalent freshwater head (m), *ρ* is the generic water density (kg/m^3^), which depends on the salt concentration *c* (kg/m^3^), *ρ*_*f*_ is the freshwater density (kg/m^3^), *ρ*_*s*_ is the sink and source density (kg/m^3^), *q*_*s*_ is the volumetric flow rate per unit volume, representing sink and source terms (1/day), *θ* is the porosity, *S*_*s*_ is the specific elastic storage (1/m), and **K** is the hydraulic conductivity tensor (m/day).

The second PDE is the solute‐transport equation, which reads (Bedekar et al. 2016):

(2) $$\frac{\partial c}{\partial t}=\nabla \left(D\nabla c\right)-\nabla \left(vc\right)-\frac{q_{s}}{\theta }c_{s}$$

where ***v*** is the pore velocity vector (m/day), calculated as v=−K∇h/θ through Darcy's law, and **D** is the hydrodynamic dispersion tensor (m^2^/day). In this study, no mechanical dispersion is considered, so that **D** is a diagonal matrix that accounts solely for chemical diffusion and is obtained by multiplying the effective molecular diffusion coefficient D* by the 3×3 identity matrix.

Hence the simulation of variable density flow is based on the solution of two PDEs (Equations 1 and 2), which are “coupled” through the two dependent variables *h* and *c*. The solution proposed here relies on an FD discretization over the aquifer domain, which transforms the two PDEs into the two following systems of ordinary differential equations (ODEs):

(3) $$\Lambda \cdot h+f=\beta \cdot \frac{dh}{\mathrm{dt}}$$

(4) $$\Lambda^{\prime }\cdot c+f^{\prime }=\beta^{\prime }\cdot \frac{dc}{\mathrm{dt}}$$

In Equations 3 and 4, **h** and **c** are the vectors of head and concentration at the model cells, **Λ** and **β** are the stiffness and the capacity matrices for the hydraulic head, respectively, **Λ**′, **β**′ are the stiffness and the capacity matrices for the concentration, respectively, and **f** and **f**′ represent generic sink and source terms for water and salt, respectively. The two ODE systems (Equations 3 and 4) are solved by discretizing the time‐derivatives at the right‐hand side by finite differences, that is:

(5) $$\frac{dh}{\mathrm{dt}}≅\frac{h_{t+∆t}-h_{t}}{∆t}$$

(6) $$\frac{dc}{\mathrm{dt}}≅\frac{c_{t+∆t}-c_{t}}{∆t}$$

where Δ*t* represents a time step. Following an implicit scheme, the estimation of **h** and **c** at the generic time *t* + Δ*t*, given their values at time *t*, is obtained by solving the following systems of equations:

(7) $$\left(\frac{\beta }{∆t}-\Lambda \right)\cdot h_{t+∆t}=\frac{\beta }{∆t}\cdot h_{t}+f$$

(8) $$\left(\frac{\beta^{\prime }}{∆t}-\Lambda^{\prime }\right)\cdot c_{t+∆t}=\frac{\beta^{\prime }}{∆t}\cdot c_{t}+f^{\prime }$$

which can be expressed in a simpler form as:

(9) $$A\cdot h_{t+∆t}=b$$

(10) $$A^{\prime }\cdot c_{t+∆t}=b^{\prime }$$

In Equations 9 and 10, **A** and **A′** are square matrices of size *N* equal to the number of FD cells, and **b** and **b′** are *N* × 1 column vectors. The systems (Equations 9 and 10) are generally non‐linear and coupled to one another due to the dependency of **A** and **b** on **c**_*t*+Δ*t*_, and the dependency of **A′** and **b′** on **h**_*t*+Δ*t*_. After imposing the prescribed initial and boundary conditions for the flow and the transport models, their solution is tackled by devising an iterative Picard procedure. In essence, the system flow component (Equation 9) is solved using first the concentration evaluated at the previous time step **c**_*t*_ to calculate **A** and **b**. The obtained head field **h**_*t*+Δ*t*_ is then used to update the velocity field using Darcy's law to calculate **A′** and **b′**, for the transport solution (Equation 10). The obtained concentration field **c**_*t*+Δ*t*_ is next used to update the density field, which is fed back into the system (Equation 9). This process is repeated iteratively until numerical convergence is reached for both **h**_*t*+Δ*t*_ and **c**_*t*+Δ*t*_. FD discretization details are presented in the Appendix A section.

### Proper Orthogonal Decomposition

The POD approach relies upon converting a system of linear equations of size *N* to a smaller system of linear equations of size *R* (*R* ≤ *N*). To apply this method, the use of orthogonal basis functions is essential. The main concept behind the POD is to separate, or decompose, the spatial and temporal behaviors of the system. The basis functions represent the spatial nature of the system, which depends on its spatial properties and are key elements for reducing the system's complexity. The temporal components can be calculated once the basis functions are identified. This decomposition is mathematically formulated by the Karhunen–Loève theorem (Newman 1996), according to which, the hydraulic head *h*(*x*,*y*,*z*,*t*) and the concentration *c*(*x*,*y*,*z*,*t*) are expressed as (Vermeulen et al. 2004):

(11) $$h\left(x,y,z,t\right)=h_{0}\left(x,y,z\right)+\sum_{k=1}^{\infty }U_{k}\left(x,y,z\right)H_{k}\left(t\right)$$

(12) $$c\left(x,y,z,t\right)=c_{0}\left(x,y,z\right)+\sum_{k=1}^{\infty }W_{k}\left(x,y,z\right)C_{k}\left(t\right)$$

where *h*_0_ and *c*_0_ are “steady‐state” functions, *H*_*k*_(*t*) and *U*_*k*_(*x*, *y*, *z*) are time and space‐dependent functions for the hydraulic head, and *C*_*k*_(*t*) and *W*_*k*_(*x*, *y*, *z*) are time and space‐dependent functions for the concentration, respectively. Both sets of *U*_*k*_ and *W*_*k*_ space‐dependent functions are orthogonal (i.e., the spatial integral of their product equals zero). By selecting a finite number *R* of representative basis functions, and discretizing *h* and *c* on the *N* cells of the FD grid, the hydraulic head and concentration vectors may be approximated as (Li et al. 2013):

(13) $$h\left(t\right)=h_{0}+\sum_{k=1}^{R}U_{k}\cdot H_{k}\left(t\right)=h_{0}+U\cdot H_{t}$$

(14) $$c\left(t\right)=c_{0}+\sum_{k=1}^{R}W_{k}\cdot C_{k}\left(t\right)=c_{0}+W\cdot C_{t}$$

where U and W are *N* × *R* matrices, whose columns include the basis function values at the FD grid cells, whereas $H_{t}$ and $C_{t}$ are *R* × 1 column vectors including the time functions H_*k*_(*t*) and C_*k*_(*t*), where *k* = 1, 2, *R*.

Capturing the spatial basis functions can be achieved by constructing “snapshot sets,” that is, system states (h and c), evaluated using the full‐scale model at different times and for different dynamic simulation scenarios for sink/source terms and/or boundary conditions. For example, in aquifers subject to pumping and spatio‐temporally variable groundwater recharge, these scenarios may include different well locations, extraction rates and schedules, and different groundwater recharge patterns. Notably, the quantity of snapshots and the selection of representative simulation scenarios is crucial as they affect the quality of the basis functions, which in turn accuracy of the POD model. If “unseen” pumping schemes and recharge patterns fall within the range of behaviors represented by these scenarios, the ROM is likely to closely reproduce the results of the full‐scale model. Otherwise, new scenarios are required and the ROM will need to be updated to include the corresponding snapshot sets. This flexibility is a key feature of the POD approach.

In this work, a “snapshot” represents the *N* × 1 vectors h and c at a specific time. With *T* time steps, the collection of snapshots over time creates an *N* × *T* matrix for both *h* and *c*. For *Q* simulations, representing different dynamic simulation scenarios, snapshots are collected for each scenario, resulting in as many *N × T* matrices, which are then concatenated horizontally into the two *N × M* matrices (*M* = *Q*·*T*) **H**_*obs*_ and **C**_*obs*_.

Once these matrices are assembled, the basis function can be derived using the SVD algorithm. The SVD (Golub and Kahan 1965), a widely used method for identifying important patterns and structures within data, relies on extracting and compressing information from data into linear combinations of orthogonal eigenfunctions multiplied by low‐order time‐dependent weights (Golub and Van Loan 2013). With the SVD, the matrices **H**_*obs*_ and **C**_*obs*_, are factorized as:

(15) $$H_{\mathrm{obs}}=U_{h}\cdot \sum_{h}\cdot {V_{h}}^{T}$$

(16) $$C_{\mathrm{obs}}=U_{c}\cdot \sum_{c}\cdot {V_{c}}^{T}$$

In Equations 15 and 16, $U_{h}$ and $U_{c}$ are orthonormal *N* × *N* matrices (with rows and columns forming a set of orthogonal unit vectors), $\sum_{h}$ and $\sum_{c}$ are *N* × *M* diagonal matrices containing “singular values,” and $V_{h}$ and $V_{c}$ are orthonormal *M* × *M* matrices. $U_{h}$ and $U_{c}$ contain the eigenvectors of the matrices $H_{\mathrm{obs}}\cdot {H_{\mathrm{obs}}}^{T}$ and $C_{\mathrm{obs}}\cdot {C_{\mathrm{obs}}}^{T}$, respectively, whereas $V_{h}$ and $V_{c}$ contain eigenvectors of the matrices ${H_{\mathrm{obs}}}^{T}\cdot H_{\mathrm{obs}}$ and ${C_{\mathrm{obs}}}^{T}\cdot C_{\mathrm{obs}}$, respectively. The “singular” values in $\sum_{h}$ and $\sum_{c}$ are the square root of the *M* eigenvalues of ${H_{\mathrm{obs}}}^{T}\cdot H_{\mathrm{obs}}$ and ${C_{\mathrm{obs}}}^{T}\cdot C_{\mathrm{obs}}$, respectively. Ranking these eigenvalues in decreasing order allows the identification of the dominant modes of variability in the data, which is instrumental for dimensionality reduction. In practice, this is achieved by selecting the *R* larger singular values (R≤M), and since in Equations 15 and 16 $U_{h}$ and $U_{c}$ include spatial components and $\sum_{h}\cdot {V_{h}}^{T}$ and $\sum_{c}\cdot {V_{c}}^{T}$ include time components of the head and concentration snapshots, the basis function matrices U and W (Equations 13 and 14) are formed by selecting the first *R* columns of $U_{h}$ and $U_{c}$.

### Variable Density Flow Reduced Order Model

The FD model reduction is achieved by substituting the head and concentration vectors h and c given by Equations 13 and 14 into the ODE systems (Equations 3 and 4), and pre‐multiplying them by the transpose matrices $U^{T}$ and $W^{T}$, respectively. This leads to expressing these in terms of the *R* time functions $H_{k}\left(t\right)$ and $C_{k}\left(t\right)$ (k=1,…,R). Using an FD discretization for these as in Equations 5 and 6, and adopting implicit schemes as in Equations 7 and 8, leads ultimately to transforming the coupled systems (Equations 9 and 10) into:

(17) $$\left[U^{T}\cdot A\cdot U\right]\cdot H_{t+∆t}=U^{T}\cdot b$$

(18) $$\left[W^{T}\cdot A^{\prime }\cdot W\right]\cdot C_{t+∆t}=W^{T}\cdot b^{\prime }$$

Equations 17 and 18 represent a system of 2⋅R equations in 2⋅R unknowns, which needs to be solved at each time step. The computational gain of this approach stems from reducing the number of unknowns from 2⋅N in Equations 9 and 10 down to 2⋅R. Once the H and C solutions are calculated, the hydraulic head and the concentration vectors h and c are estimated using Equations 13 and 14.

**Figure 1:**
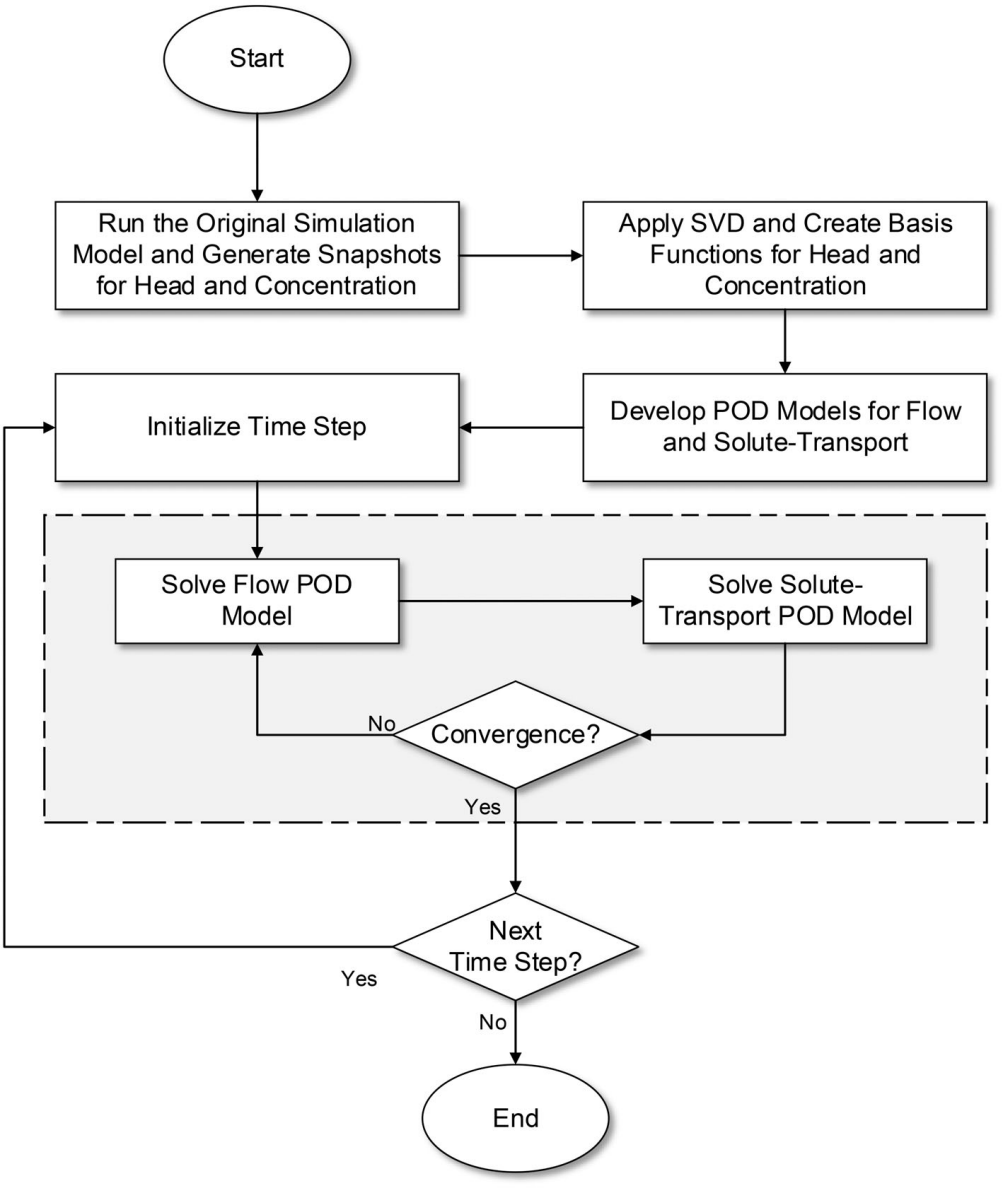
Flowchart for variable‐density flow reduced order model.

The ROM framework is described in Figure 1. The initial step consists of the compilation of the snapshot datasets **H**_*obs*_ and **C**_*obs*_. This step relies on the use of a full‐scale variable density‐flow simulation model, which, from a computational perspective, is the most expensive part of the process. Next, an SVD algorithm is applied to reconstruct the basis function matrices U and W, which are then applied to solve the reduced order systems (Equations 17 and 18).

Similar to the full‐scale systems (Equations 9 and 10), the reduced order systems (Equations 17 and 18) are nonlinear and coupled to one another due to the dependencies of **A** and **b** on **c**_*t*+Δ*t*_, and **A′** and **b′** on **h**_*t*+Δ*t*_ their solution at any given time step is thus carried out using the Picard iteration scheme described in section “Variable Density Flow Model.” Convergence is ensured through a Picard iteration scheme, where the solution is iteratively refined until the changes between successive iterations fall below a predefined tolerance (Algorithm 1). The pseudocode of the proposed model is as follows:

Algorithm 1 **POD Model for Variable‐Density Flow**

1 BEGIN.

2 // Step 1: Initialization.

3 Set *model*        // Initialize model domain, parameters, etc.

4 Get *R*          // Order of POD model.

5 Get *tolerance*    // Tolerance for Picard iteration scheme.

6 // Step 2: Compute Snapshots.

7  **for** *t* in range(*T*): // Loop through time steps.

8   **while** |**c**_t+1_ − **c**_t+1,old_| > *tolerance*   // Picard iteration for snapshots.

9     get **h**_t+1_ by Equation 9    // Compute head at t + 1.

10     set **c**_t+1,old =_ **c**_t+1_       // Update previous concentration.

11     get **c**_t+1_ by Equation 10  // Compute concentration at t + 1.

12 // Step 3: Compute Basis Functions.

13  get **U**_h_ by Equation 15    // Compute basis functions for head.

14  get **U**_c_ by Equation 16    // Compute basis functions for concentration.

15 // Step 4: Construct POD model.

16   **for** *t* in range(*T*):      // Loop through time steps for POD mode.

17   **while** |**c**_t+1_ − **c**_t+1,old_| > *tolerance*  // Picard iteration for POD model.

18     get **H**_t+1_ by Equation 17   // Compute head time function at t + 1.

19     get **h**_t+1_ by Equation 13   // Compute head at t + 1.

20     set **c**_t+1,old_ = **c**_t+1_      // Update previous concentration.

21     get **C**_t+1_ by Equation 18   // Compute concentration time function at t + 1.

22     get **c**_t+1_ by Equation 14   // Compute concentration at t + 1.

23 // Step 5: Results Output.

24 Print **h** and **c**   // Print final head and concentration matrices.

25 END.

It is important to point that the snapshot datasets **h**_*obs*_ and **c**_*obs*_ that are used to reconstruct the basis function matrices U and W should not necessarily be assembled using the same FD variable density flow model (Equations 9 and 10) that is then used to construct the reduced order systems of Equations 17 and 18. Indeed, it is possible to use any other variable density flow model, so long as this complies with the same spatial and temporal discretization, input parameters, initial and boundary conditions, and sources and sink inputs as the FD variable density flow model presented in section “Variable Density Flow Model.”

### Numerical Experiments

**Figure 2:**
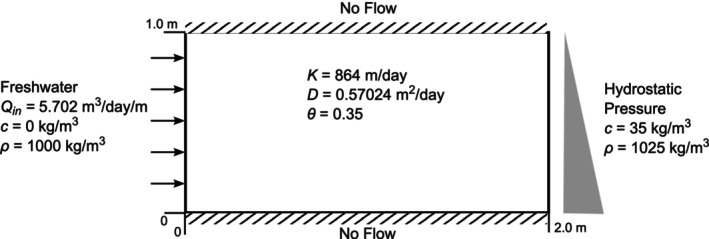
Henry's benchmark problem: vertical cross‐section of the model domain including flow and transport boundary conditions.

Henry's problem is a classical benchmark for testing SWI models. This problem (Henry 1964) considers a confined porous medium characterized by a 2×1 (m×m) rectangular domain, whose vertical cross‐section is depicted in Figure 2. Flow‐wise, both the top and bottom boundaries of the domain are assumed to be impermeable. At the left boundary, a constant and uniform specified flux is assumed, mimicking inland boundary conditions. At the right boundary, a uniform and constant head is prescribed, representing a hydrostatic pressure distribution at the seashore interface. Top and bottom boundaries are considered impermeable also transport‐wise. The salt concentration is fixed at 35 g/L at the right boundary, and to zero at the left boundary, signifying influx of freshwater.

Henry (1964) provided a semi‐analytical solution for this problem, laying the foundation for understanding SWI in coastal aquifers. Henry's solution assumes salt migration is driven solely by advection and diffusion processes, excluding hydrodynamic dispersion due to heterogeneity in the hydraulic conductivity. The solution describes a steady‐state density stratification, with denser salt water infiltrating below the lighter freshwater, forming a characteristic sea water wedge that mediates the advection of freshwater entering the aquifer from the left boundary with the inland diffusion of sea water originating from the right boundary, which modifies the water density.

The model parameters adopted in this work are those taken from (Guo and Langevin 2002). The domain is discretized with a uniform 2D grid of 200×100 grid blocks (*N* = 20,000), each of size 0.02 m × 0.02 m. The effective molecular diffusion D* is assumed to be equal to 0.57024 m^2^/d and the porosity θ is 0.35.

While the Henry's problem considers steady‐state conditions, the numerical tests presented in the following are conducted in transient state, with steady‐state achieved at “large” time, which, for the problem at hand, is practically reached at *t* = 500 min. In all simulations, initial conditions (at *t* = 0) assume that the freshwater hydraulic head h is equal to 1.0 m and the solute concentration C is equal to zero throughout the domain.

The snapshot datasets **h**_*obs*_ and **c**_*obs*_ (section “Proper Orthogonal Decomposition”) are assembled by using the h and C model output at each time step, with ∆t=1min, so that both matrices have a size N×M of 20,000×500.

#### Scenario 1: Homogenous System

The first testing scenario is based on the original Henry's problem, in which the system is assumed to be homogeneous with a hydraulic conductivity K of 864 m/d. Four different solvers are applied and compared: (a) FD: the developed FD full‐scale model (Equations 9 and 10); (b) FD‐POD: a POD‐based reduced order FD model (Equations 17 and 18) with snapshots generated using the FD full‐scale model; (c) MF6: the FV‐based MODFLOW 6 code; and (d) FD‐POD‐MF6: a POD‐based reduced order FD model (Equations 17 and 18) with snapshots generated using MODFLOW 6.

**Figure 3:**
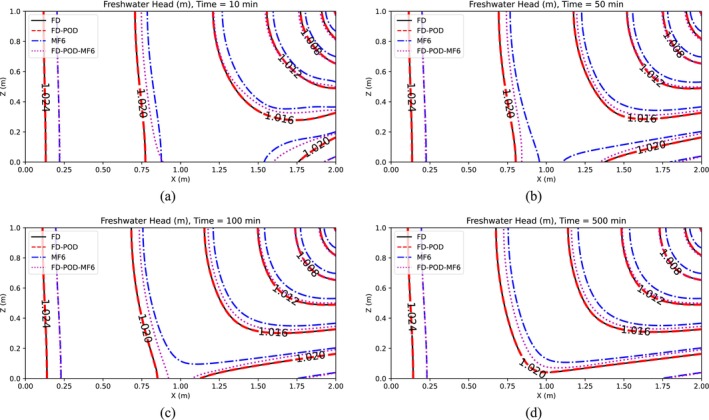
Freshwater hydraulic head for Henry's Problem (Scenario 1), after (a) 10 min, (b) 50 min, (c) 100 min, and (d) 500 min.

**Figure 4:**
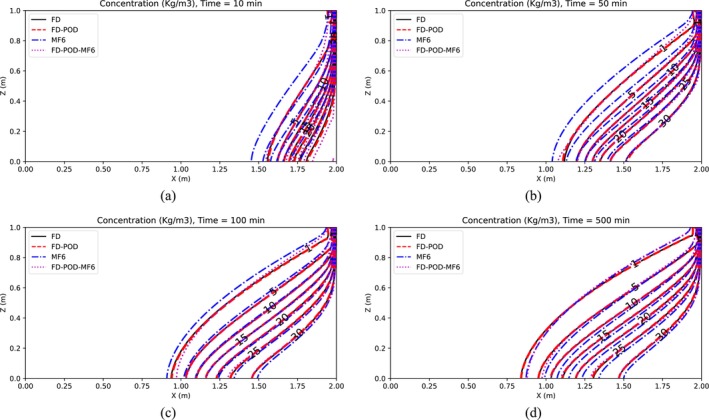
Concentrations for Henry's Problem (Scenario 1), after (a) 10 min, (b) 50 min, (c) 100 min, and (d) 500 min.

Simulation results are presented in Figures 3 and 4, which display maps of the hydraulic head and the solute concentration, respectively, for the case in which *R* = 10 is selected.

Results in Figures 3 and 4 show that, at all assessed times (10, 50, 100, 500 min), the FD model (Section “Variable Density Flow Model”) produces results that are close but do not perfectly match MF6's results, which are considered here as the benchmark solution. This is not surprising as the two models adopt different numerical schemes. The freshwater head from the FD and POD‐based FD model match very closely. In addition, it is observed that the POD‐based FD model obtained using snapshots generated by MF6, produces freshwater head distributions that match MF6's better than when these snapshots are generated with the FD model. As for salt concentration, results from all four models match generally well particularly at larger times, with MF6 results being slightly higher than other models, particularly at lower times.

Table 1 summarizes model comparisons from the application of the POD‐based RO approaches for increasing values of *R*. These comparisons are conducted in terms of the coefficient of determination *r* ^2^ and the relative root mean squared error (RRMSE) calculated for freshwater head and concentration by considering the outputs of the full‐scale models versus those from the POD‐based ROMs at *t* = 500 min. Results show a slight general improvement for values of *R* that increase from 10 to 50, indicating that selecting *R* equal to 10 is acceptable in this case. In terms of computational gain, the reduction in model size is observed to lead to a drastic reduction in calculation time, which results at least one order of magnitude smaller. Because with the same *R* for flow and solute transport model, not equal precision is achieved, which is likely due the complexity of the solute transport PDE, in comparison with the flow PDE.

**Table 1:** Comparison of POD Models for the Original Henry's Problem at *t* = 500 min

		Freshwater Head (m)		Concentration (kg/m^3^)	
Snapshot Generation	POD Rank *R*	*r* ^2^	RRMSE	*r* ^2^	RRMSE
FD	10	0.9999988	10.12 × 10^−6^	0.9999639	7.33 × 10^−6^
	20	0.9999991	8.47 × 10^−6^	0.9999709	6.65 × 10^−6^
	50	0.9999992	8.39 × 10^−6^	0.9999716	6.53 × 10^−6^
MF6	10	0.9913467	8.37 × 10^−4^	0.9956631	7.94 × 10^−4^
	20	0.9914066	8.34 × 10^−4^	0.9958612	7.72 × 10^−4^
	50	0.9918227	8.14 × 10^−4^	0.9962977	7.31 × 10^−4^

#### Scenario 2: Heterogeneous System

**Figure 5:**
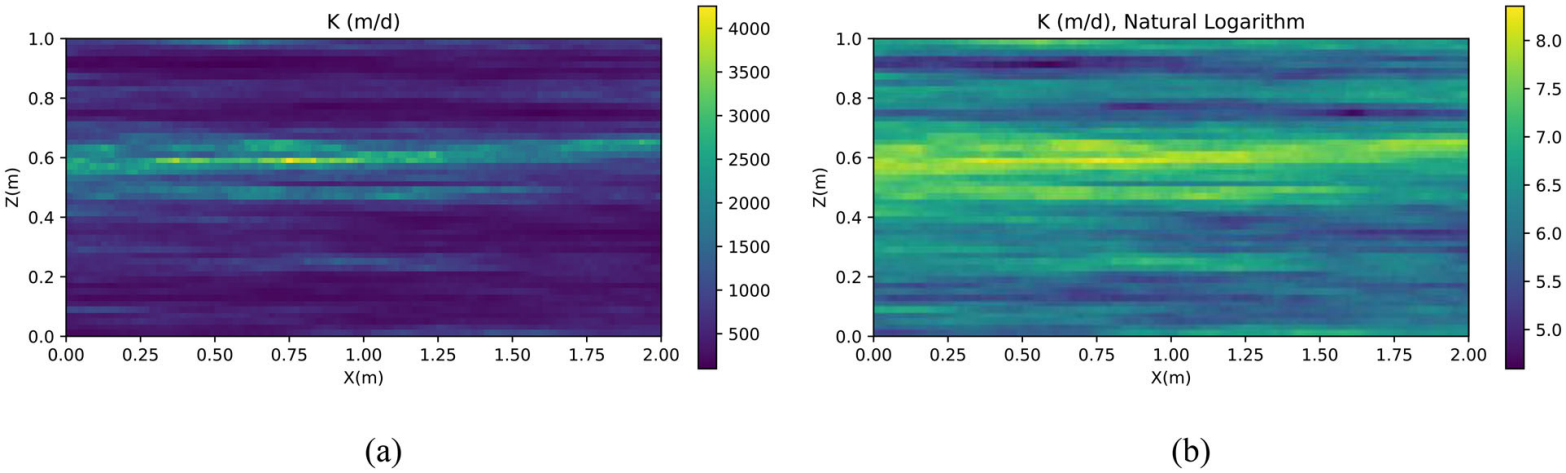
Heterogeneous *K* field, (a) exact values, (b) natural logarithmic values.

**Figure 6:**
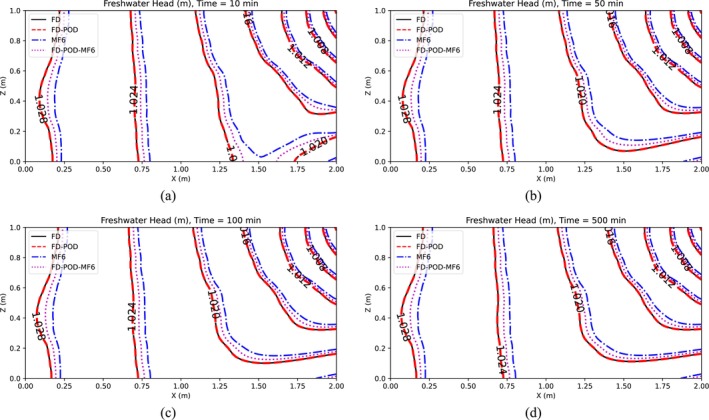
Freshwater hydraulic head for Henry's Problem in the case of a heterogeneous aquifer, after (a) 10 min, (b) 50 min, (c) 100 min, and (d) 500 min.

**Figure 7:**
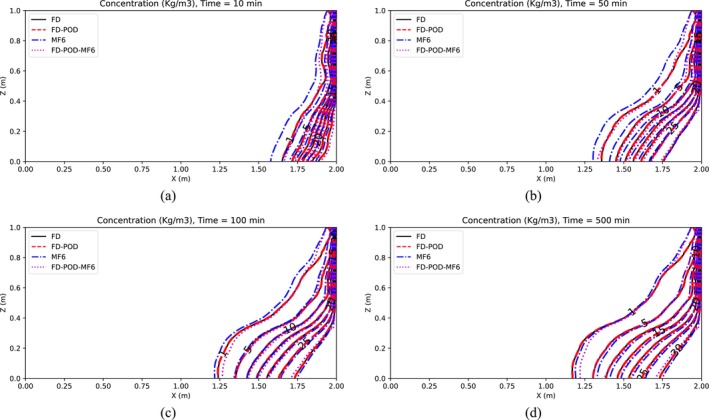
Concentrations for heterogeneous Henry's Problem, after (a) 10 min, (b) 50 min, (c) 100 min, and (d) 500 min.

To test the POD‐base model reduction approaches under more complex conditions, a heterogeneous spatial distribution of the hydraulic conductivity *K* is hypothesized (Figure 5). This is generated as a single realization of stochastic log‐normal process characterized by an average $\mu_{\ln K}=6.761$ (i.e., ln(864)) and an anisotropic exponential covariance function with variance $\sigma_{\ln K^{2}}=1$, and correlation scales $\lambda_{x}=2\;m$ and $\lambda_{z}=0.1\;m$ in the horizontal and vertical directions. As in Scenario 1, the solutions from four different models are compared. Figures 6 and 7 display model results for freshwater head and concentration, respectively. These results show a general good agreement for such a complex stochastic *K* field. The FD model and the FD‐POD model with *R* = 10 show *h* and *C* outputs that are always practically the same but differ, however slightly, from those calculated with MF6 and the POD reduced model constructed using MF6 snapshots with *R* = 10. Such a difference is generally more pronounced for the *h* field than for the *C* field.

Table 2 displays *r* ^2^ and RRMSE calculated for *h* and *C* at *t* = 500 min by comparing the POD‐based reduced order model outputs to those from the full‐scale models. Results show the FD‐POD model matches the FD model results quite well already with *R* equal to 10. The FD‐POD model with snapshots generated by MF6 does not perform as well in terms of accuracy with respect to MF6, indicating that values of *R* of the order of 50 or larger are preferable.

**Table 2:** Comparison of POD Models for the Heterogeneous Henry's Problem at *t* = 500 min

		Freshwater Head (m)		Concentration (kg/m^3^)	
Snapshot Generation	POD Rank *R*	*r* ^2^	RRMSE	*r* ^2^	RRMSE
FD	10	0.99999977	6.04 × 10^−6^	0.9997752	1.25 × 10^−5^
	20	0.99999983	5.12 × 10^−6^	0.9999181	1.18 × 10^−5^
	50	0.99999984	5.01 × 10^−6^	0.9999343	1.06 × 10^−5^
MF6	10	0.99589707	9.45 × 10^−4^	0.9895655	13.16 × 10^−4^
	20	0.99687873	8.29 × 10^−4^	0.9961524	7.99 × 10^−4^
	50	0.99706948	8.07 × 10^−4^	0.9973246	6.66 × 10^−4^

Considering the more complex heterogeneous Henry's problem, the challenges associated with accurately capturing SWI dynamics become apparent. Heterogeneity introduces irregularities in the aquifer properties, requiring a larger number of POD components to accurately represent the spatial patterns. Despite this, the FD‐POD approach demonstrates promising results, with high *r* ^2^ values and relatively low RRMSE values for both the freshwater head and concentration compared with the MF6 models.

Overall, these results highlight an interesting feature: by generating a set of snapshots with MF6, a POD model can be constructed as a faster surrogate, replacing the original MF6 model. This capability significantly reduces computational costs, making the FD‐POD approach an attractive option for SWI simulations.

## Discussion

The essence of the POD model lies in the reduced computational cost that can be achieved by retrieving the approximated models (Equations 13 and 14), which ultimately allows for the solution of the reduced‐order systems (Equations 17 and 18) instead of the full‐scale systems (Equations 9 and 10). The ROM accuracy depends ultimately on the quality of basis functions stored in the matrices **H** and **W**. Such a quality depends on the representativeness of the boundary condition scenarios considered in the calculation of the snapshot sets, referred to as **H**_obs_ and **C**_obs_, and the reduced number *R* of orthonormal basis functions selected to approximate the state functions *h*(*x*,*y*,*z*,*t*) and *c*(*x*,*y*,*z*,*t*). While the selection of *R* can be somehow “automated” by selecting the higher eigenvalues (e.g., by selecting only those exceeding a prescribed threshold fraction [e.g., 0.01] of the maximum eigenvalue), the selection of representative boundary condition scenarios, depends on the complexity of the boundary conditions themselves. Nonetheless when addressing the balance between efficiency and precision, of course, one cannot run the full‐scale model for any unseen boundary condition set as this would basically void the computational gain of the ROM approach. However, it seems reasonable to assess the accuracy of the POD‐based model through comparison with a smaller set of full‐scale simulations. For example, if the ROM was used to substitute a full‐scale model in a simulation‐optimization framework, the smaller set of validating simulations could be chosen from the optimal sets of solutions or in the “surrounding” them. If the POD model is deemed to be sufficiently accurate, then it can be trusted for further simulations. Regarding “model complexity,” the tests presented in our article generally show that larger *R* values are needed in the case of highly heterogeneous systems, but this does not appear to reduce efficiency significantly. In terms of the size of snapshot sets, adequate values should be determined based on the range of conditions (e.g., pumping rates, boundary conditions) captured in the simulations. Note that in our test only one scenario was tested (boundary conditions are constant and constant), and additional snapshots should be considered, for example, if also effects of pumping wells on SWI were to be considered.

## Conclusions

In this study, a novel approach has been introduced to merge POD with FD methods to address the computational challenges associated with variable‐density flow in coastal aquifers. Focusing on simulating SWI, particularly addressing Henry's problem in both homogeneous and heterogeneous media, the aim was to enhance computational efficiency while maintaining accuracy compared with full‐scale models.

The significance of combating SWI challenges in coastal aquifers cannot be overstated. The proposed FD‐POD approach emerged as a potent tool for groundwater management, leveraging reduced‐order models to navigate the complexities of variable‐density flow dynamics. By emphasizing the balance between efficiency and precision, the approach offers promising solutions for sustainable coastal groundwater resource management.

Benchmarking the FD‐POD model against established methods like MF6 reveals compelling findings. The model yields results comparable to traditional methods, showcasing its efficacy in capturing the essential dynamics of density‐dependent flow and solute transport. Moreover, while minor discrepancies exist, the overall computational gains outweigh these, emphasizing the model's ability to strike a balance between efficiency and accuracy.

The strengths of the POD‐based reduced‐order model are manifold. Its computational efficiency, evidenced by significant reductions in model size and calculation time, makes it a cost‐effective solution for simulating SWI in coastal aquifers. Moreover, its accuracy ensures reliable simulations while simplifying the computational complexity of variable‐density flow dynamics. For instance, when a model needs to be run several times, such as in optimization‐simulation approaches, the POD presents a unique advantage. A set of snapshots could be generated with MF6 to create basis functions, and subsequently, a POD model could be constructed as a faster surrogate model, replacing the original MF6 model.

While FD‐POD may provide a computationally efficient and accurate surrogate model, its effectiveness hinges on the diversity and representativeness of the snapshots used in its construction. For example, in simulation‐optimization applications for groundwater management in coastal aquifers, it is crucial to generate snapshots across a broad spectrum of potential well configurations, pumping rates, and schedules. This ensures that the ROM can accurately capture the system's behavior under various operating conditions.

Practically, the model facilitates rapid decision‐making in coastal groundwater management, allowing for timely responses to changing environmental conditions. Its adoption supports sustainable resource management by assessing the impact of SWI on water quality and aquifer sustainability. Moreover, it enhances decision support systems, aiding in the development of effective strategies to mitigate intrusion and protect freshwater resources.

Looking forward, several avenues for future research and development emerge. Refinement of model calibration processes could enhance accuracy under varying hydrogeological conditions. Integration of uncertainty quantification techniques could assess the impact of uncertainties on model predictions. Validation in diverse real world scenarios is imperative for understanding performance under complex real world conditions. Integration with decision support systems and exploration of multi‐objective optimization techniques could further enhance practical utility and identify optimal solutions for coastal aquifer management.

In addressing these limitations and exploring future research avenues, the FD‐POD approach stands poised to evolve and meet the evolving challenges of groundwater management and sustainability in coastal regions. Its potential to revolutionize SWI simulation in coastal aquifers is evident, offering a comprehensive framework for efficient and accurate decision‐making in resource management.

## Data Availability

The data that support the findings of this study are available from the corresponding author upon reasonable request.

## Appendix A

### Finite Difference Discretization

Considering density‐dependent flow PDE as:

(A1)
$$\begin{array}{ll} & \frac{\partial }{\partial x}\left(\rho K^{x}\frac{\partial h}{\partial x}\right)+\frac{\partial }{\partial y}\left(\rho K^{y}\frac{\partial h}{\partial y}\right)\\ & \;+\frac{\partial }{\partial z}\left(\rho K^{z}\left\{\frac{\partial h}{\partial z}+\frac{\rho -\rho_{f}}{\rho_{f}}\right\}\right)\\ & \;=\rho S_{s}\frac{\partial h}{\partial t}+\theta \frac{\partial \rho }{\partial c}\frac{\partial c}{\partial t}-\rho_{s}q_{s} \end{array}$$

By applying an FD spatial discretization Equation A1 becomes:

(A2)
$$\begin{array}{ll} & \frac{1}{\Delta x}\left({\left[\rho K^{x}\frac{\partial h}{\partial x}\right]}_{i+0.5}-{\left[\rho K^{x}\frac{\partial h}{\partial x}\right]}_{i-0.5}\right)\\ & \;+\frac{1}{\Delta y}\left({\left[\rho K^{y}\frac{\partial h}{\partial y}\right]}_{j+0.5}-{\left[\rho K^{y}\frac{\partial h}{\partial y}\right]}_{j-0.5}\right)\\ & \;+\frac{1}{\Delta z}\left({\left[\rho K^{Z}\left\{\frac{\partial h}{\partial z}+\frac{\rho -\rho_{f}}{\rho_{f}}\right\}\right]}_{k+0.5}\right.\\ & \;\times \left.-{\left[\rho K^{Z}\left\{\frac{\partial h}{\partial z}+\frac{\rho -\rho_{f}}{\rho_{f}}\right\}\right]}_{k-0.5}\right)\\ & \;=\rho S_{s}\frac{\partial h}{\partial t}+\theta \frac{\partial \rho }{\partial c}\frac{\partial c}{\partial t}-\rho_{s}q_{s} \end{array}$$

where *i*, *j*, and *k* represent cell indices in *x*, *y*, and *z* directions, respectively. The index *i* + 0.5 and *i* − 0.5 represent a property related to the cell *i* to *i* + 1 and *i* − 1 to *i*, respectively. Similarly, *j* + 0.5, *j* − 0.5, *k* + 0.5, and *k* − 0.5 are defined. *K* ^*x*^, *K* ^*y*^, and *K* ^*z*^ represent hydraulic conductivity in *x*, *y*, and *z* directions. Multiplying both sides by the cell volume and using an FD approximation in time with an implicit approach yield:

(A3)
$$\begin{array}{ll} & \Delta y\Delta z\rho_{i+0.5,j,k}K_{i+0.5,j,k}^{x}\frac{h_{i+1,j,k}^{t+1}-h_{i,j,k}^{t+1}}{\Delta x}\\ & \;-\Delta y\Delta z\rho_{i-0.5,j,k}K_{i-0.5,j,k}^{x}\frac{h_{i,j,k}^{t+1}-h_{i-1,j,k}^{t+1}}{\Delta x}\\ & \;+\Delta x\Delta z\rho_{i,j+0.5,k}K_{i,j+0.5,k}^{y}\frac{h_{i,j+1,k}^{t+1}-h_{i,j,k}^{t+1}}{\Delta y}\\ & \;-\Delta x\Delta z\rho_{i,j-0.5,k}K_{i,j-0.5,k}^{y}\frac{h_{i,j,k}^{t+1}-h_{i,j-1,k}^{t+1}}{\Delta y}\\ & \;+\Delta x\Delta y\rho_{i,j,k+0.5}K_{i,j,k+0.5}^{z}\left(\frac{h_{i,j,k+1}^{t+1}-h_{i,j,k}^{t+1}}{\Delta z}\right.\\ & \;\left.+\frac{\rho_{i,j,k+0.5}-\rho_{f}}{\rho_{f}}\right)-\Delta x\Delta y\rho_{i,j,k-0.5}K_{i,j,k-0.5}^{z}\\ & \;\times \left(\frac{h_{i,j,k}^{t+1}-h_{i,j,k-1}^{t+1}}{\Delta z}+\frac{\rho_{i,j,k-0.5}-\rho_{f}}{\rho_{f}}\right)\\ & \;=\left(\Delta x\Delta y\Delta z\right)\left(\rho S_{s}\frac{h_{i,j,k}^{t+1}-h_{i,j,k}^{t}}{\Delta t}+\theta E\frac{\partial c}{\partial t}-\rho_{s}q_{s}\right) \end{array}$$

where *t* is the time step index, *E = ∂ρ/∂c* = 0.7143 (Guo and Langevin 2002) and Δ*x*, Δ*y*, and Δ*z* are the dimensions of each cell in *x*, *y*, and *z* directions. Equation A3 can be expressed as:

(A4)
$$\begin{array}{ll} & d_{1}h_{i-1,j,k}^{t+1}+d_{2}h_{i+1,j,k}^{t+1}+d_{3}h_{i,j-1,k}^{t+1}+d_{4}h_{i,j+1,k}^{t+1}\\ & \;+d_{5}h_{i,j,k-1}^{t+1}+d_{6}h_{i,j,k+1}^{t+1}+d_{7}h_{i,j,k}^{t+1}=d_{0} \end{array}$$

where *d*_0_ to *d*_7_ are as follows:

(A5)
$$\begin{array}{ll} d_{0} & =-\frac{\Delta x\Delta y}{\rho_{f}}\rho_{i,j,k+0.5}K_{i,j,k+0.5}^{z}\left(\rho_{i,j,k+0.5}-\rho_{f}\right)\\ & \;-\frac{\Delta x\Delta y}{\rho_{f}}\rho_{i,j,k-0.5}K_{i,j,k-0.5}^{z}\left(\rho_{i,j,k-0.5}-\rho_{f}\right)\\ & \;+\left(\theta E\frac{\partial c}{\partial t}-\frac{\rho S_{s}}{\Delta t}\left(h_{i,j,k}^{t}\right)-\rho_{s}q_{s}\right)\\ & \;\times \left(\Delta x\Delta y\Delta z\right) \end{array}$$

(A6)
$$d_{1}=\frac{\Delta y\Delta z}{\Delta x}\rho_{i-0.5,j,k}K_{i-0.5,j,k}^{x}$$

(A7)
$$d_{2}=\frac{\Delta y\Delta z}{\Delta x}\rho_{i+0.5,j,k}K_{i+0.5,j,k}^{x}$$

(A8)
$$d_{3}=\frac{\Delta x\Delta z}{\Delta y}\rho_{i,j-0.5,k}K_{i,j-0.5,k}^{y}$$

(A9)
$$d_{4}=\frac{\Delta x\Delta z}{\Delta y}\rho_{i,j+0.5,k}K_{i,j+0.5,k}^{y}$$

(A10)
$$d_{5}=\frac{\Delta x\Delta y}{\Delta z}\rho_{i,j,k-0.5}K_{i,j,k-0.5}^{z}$$

(A11)
$$d_{6}=\frac{\Delta x\Delta y}{\Delta z}\rho_{i,j,k+0.5}K_{i,j,k+0.5}^{z}$$

(A12)
$$\begin{array}{ll} d_{7} & =-\left(d_{1}+d_{2}+d_{3}+d_{4}+d_{5}+d_{6}\right)\\ & \;-\frac{\rho_{i,j,k}S_{s}}{\Delta t}\left(\Delta x\Delta y\Delta z\right) \end{array}$$

When applied to each of the FD grid cells, Equation A4 produces a system of equations, which may be written in matrix form as Equation 9.

The solute transport PDE with no mechanical dispersivity is written as:

(A13)
$$\begin{array}{ll} \frac{\partial c}{\partial t} & =D\left(\frac{\partial^{2}c}{\partial x^{2}}+\frac{\partial^{2}c}{\partial y^{2}}+\frac{\partial^{2}c}{\partial z^{2}}\right)-\frac{\partial }{\partial x}\left(v^{x}c\right)\\ & -\frac{\partial }{\partial y}\left(v^{y}c\right)-\frac{\partial }{\partial z}\left(v^{z}c\right)+\frac{q_{s}c_{s}}{\theta } \end{array}$$

Using an FD method with an implicit approach Equation A13 is transformed to:

(A14)
$$\begin{array}{ll} \frac{c_{i,j,k}^{t+1}-c_{i,j,k}^{t}}{\Delta t} & =D\left(\frac{c_{i-1,j,k}^{t+1}-2c_{i,j,k}^{t+1}+c_{i+1,j,k}^{t+1}}{\Delta x^{2}}\right.\\ & \;+\frac{c_{i,j-1,k}^{t+1}-2c_{i,j,k}^{t+1}+c_{i,j+1,k}^{t+1}}{\Delta y^{2}}.\\ & \;\left.+\frac{c_{i,j,k-1}^{t+1}-2c_{i,j,k}^{t+1}+c_{i,j,k+1}^{t+1}}{\Delta z^{2}}\right)\\ & \;-\frac{v_{i+1,j,k}^{x}c_{i+1,j,k}^{t+1}-v_{i-1,j,k}^{x}c_{i-1,j,k}^{t+1}}{2\Delta x}\\ & \;-\frac{v_{i,j+1,k}^{y}c_{i,j+1,k}^{t+1}-v_{i,j-1,k}^{y}c_{i,j-1,k}^{t+1}}{2\Delta y}\\ & \;-\frac{v_{i,j,k+1}^{z}c_{i,j,k+1}^{t+1}-v_{i,j,k-1}^{z}c_{i,j,k-1}^{t+1}}{2\Delta z}\\ & \;+\frac{q_{s}c_{s}}{\theta } \end{array}$$

which can be expressed as:

(A15)
$$\begin{array}{ll} & e_{1}c_{i-1,j,k}^{t+1}+e_{2}c_{i+1,j,k}^{t+1}+e_{3}c_{i,j-1,k}^{t+1}+e_{4}c_{i,j+1,k}^{t+1}\\ & \;+e_{5}c_{i,j,k-1}^{t+1}+e_{6}c_{i,j,k+1}^{t+1}+e_{7}c_{i,j,k}^{t+1}=e_{0} \end{array}$$

where the coefficients *e*_0_ to *e*_7_ are:

(A16)
$$e_{0}=-\frac{1}{\Delta t}c_{i,j,k}^{t}-\frac{q_{s}c_{s}}{\theta }$$

(A17)
$$e_{1}=\frac{D}{\Delta x^{2}}+\frac{v_{i-1,j,k}^{x}}{2\Delta x}$$

(A18)
$$e_{2}=\frac{D}{\Delta x^{2}}-\frac{v_{i+1,j,k}^{x}}{2\Delta x}$$

(A19)
$$e_{3}=\frac{D}{\Delta y^{2}}+\frac{v_{i-1,j,k}^{y}}{2\Delta y}$$

(A20)
$$e_{4}=\frac{D}{\Delta y^{2}}-\frac{v_{i+1,j,k}^{y}}{2\Delta y}$$

(A21)
$$e_{5}=\frac{D}{\Delta z^{2}}+\frac{v_{i-1,j,k}^{z}}{2\Delta z}$$

(A22)
$$e_{6}=\frac{D}{\Delta z^{2}}-\frac{v_{i+1,j,k}^{z}}{2\Delta z}$$

(A23)
$$e_{7}=-\frac{2D}{\Delta x^{2}}-\frac{2D}{\Delta y^{2}}-\frac{2D}{\Delta z^{2}}-\frac{1}{\Delta t}$$

When applied to each FD grid cell, Equation A15 generates a system of equations, which may be written in matrix form as Equation 10.
